# On the Use of Dry-Powdered Blood

**Published:** 1873-07

**Authors:** 

**Affiliations:** Nice


*On the Use of Dry-Powdered Blood.* By DR. DE PASCALE, of Nice.

Several years ago, I published from my experience and medical practice, some observations on the very beneficial effect of warm blood taken the moment when extracted from the calf or ox, killed for general domestic use.

I mentioned at that time the cases of three invalids, not English, suffering from hæmoptysis, in whom tubercles were diagnosticated, who derived great benefit from that treatment. The quantity of blood lost by one of the abovementioned invalids was enormous; but his perseverance for two years or more in drinking daily the blood, made him well and healthy. At this present time he is walking about Nice, or attending to the business of his large establishment.

I do not wish to dwell upon the great improvement in my own general health after drinking the warm blood for about a month. One of the English doctors practicing in this place had the opportunity of verifying my improvement, and the experiment which I made, when in a state of general weakness and pallor, in consequence of suffering for many years from malarial fever, taken during the siege of Venice in 1848 and 1849.

Every one knows the history of those barbarians, who were accustomed to drink the blood of their victims at a feast after their battles; and also of those who were supported by the blood of their companions, wrecked in the *Medusa* in 1807; and of others who have been nourished in the desert by the blood of animals.

Dioscorides affirms, in his *De Medicinali Materia*, that animal blood has been used for the purpose of curing diseases; the old women adopted a similar system.

Finding among the English and American residents in Nice an unconquerable repugnance to such a remedy, the name alone having the power of producing nausea, I was obliged to disuse it. But afterwards a dim recollection of the manner in which it was administered by old medical men in my youth, made me adopt the plan of giving it in the form of dry powder.

History also relates that dry-powdered blood was used before the thirteenth century, when the quack, Jean De Gaddesden, brought it into renown.

It is easy to understand the comparative difference between the warm and the dry blood. In the first there is life with animal heat, and volatile principles, which conduce to assimilation. Notwithstanding, in the dry blood, fibrin, albumen, hæmatozin, manganatic, and ferruginous salts remain.

Between the seventeenth and eighteenth centuries the celebrated anatomist of that time (F. Buischio) found in the blood the necessary elements for the composition of every tissue of our body. At the end of the seventeenth century also was discovered one of the most important principles of the blood, that is, iron, by M. Lamey, and afterwards by Berzelius. According to Berzelius, a distinguished professor of chemistry, the blood of the ox is most similar to that of man; this, in fact, I have used in several cases of general weakness with anæmia, and in cases of chlorosis.

The blood of the ox, after being dried in a water-bath, is reduced to a very fine powder, and grated through a sieve. Dry blood can be taken for any length of time, being almost tasteless, and no repugnance is likely to be felt, as is often the case with raw meat. It can be taken as any common powder, mixed with soups, milk, marmalade, chocolate, or enclosed in a wafer.

In two cases I have given the powdered blood under the name of nutritive powders, mixed with a small quantity of pepsine; choosing that name lest ladies, startled by one more precise, might have difficulty in taking the medicine.

The quantity to be taken may vary according to the age, sex, or the state of health and digestive power of the patient. In general, I begin with thirty grains, which is increased according to circumstances; but quantity must be left to the discretion of the physician who prescribes.—*Med. Press and Circular.*

